# Power and Predictive Accuracy of Polygenic Risk Scores

**DOI:** 10.1371/journal.pgen.1003348

**Published:** 2013-03-21

**Authors:** Frank Dudbridge

**Affiliations:** Faculty of Epidemiology and Population Health, London School of Hygiene and Tropical Medicine, London, United Kingdom; Queensland Institute of Medical Research, Australia

## Abstract

Polygenic scores have recently been used to summarise genetic effects among an ensemble of markers that do not individually achieve significance in a large-scale association study. Markers are selected using an initial training sample and used to construct a score in an independent replication sample by forming the weighted sum of associated alleles within each subject. Association between a trait and this composite score implies that a genetic signal is present among the selected markers, and the score can then be used for prediction of individual trait values. This approach has been used to obtain evidence of a genetic effect when no single markers are significant, to establish a common genetic basis for related disorders, and to construct risk prediction models. In some cases, however, the desired association or prediction has not been achieved. Here, the power and predictive accuracy of a polygenic score are derived from a quantitative genetics model as a function of the sizes of the two samples, explained genetic variance, selection thresholds for including a marker in the score, and methods for weighting effect sizes in the score. Expressions are derived for quantitative and discrete traits, the latter allowing for case/control sampling. A novel approach to estimating the variance explained by a marker panel is also proposed. It is shown that published studies with significant association of polygenic scores have been well powered, whereas those with negative results can be explained by low sample size. It is also shown that useful levels of prediction may only be approached when predictors are estimated from very large samples, up to an order of magnitude greater than currently available. Therefore, polygenic scores currently have more utility for association testing than predicting complex traits, but prediction will become more feasible as sample sizes continue to grow.

## Author Summary

Recently there has been much interest in combining multiple genetic markers into a single score for predicting disease risk. Even if many of the individual markers have no detected effect, the combined score could be a strong predictor of disease. This has allowed researchers to demonstrate that some diseases have a strong genetic basis, even if few actual genes have been identified, and it has also revealed a common genetic basis for distinct diseases. These analyses have so far been performed opportunistically, with mixed results. Here I derive formulae based on the heritability of disease and size of the study, allowing researchers to plan their analyses from a more informed position. I show that discouraging results in some previous studies were due to the low number of subjects studied, but a modest increase in study size would allow more successful analysis. However, I also show that, for genetics to become useful for predicting individual risk of disease, hundreds of thousands of subjects may be needed to estimate the gene effects. This is larger than most existing studies, but will become more common in the near future, so that gene scores will become more useful for predicting disease than has appeared to date.

## Introduction

Although individually significant markers in genome-wide association scans (GWAS) explain limited heritability of complex traits, evidence has been accruing that a considerable proportion of phenotypic variation can be explained by the ensemble of markers not achieving significance. Thus, while most of the specific genes underlying complex traits have yet to be identified, it is likely that many are represented on current genotyping products and specific identification is largely a matter of study size [1]. Polygenic score analysis has recently generated much interest for assessing the explanatory power of an ensemble of markers. A GWAS is conducted on an initial training sample, and the markers are ranked by their evidence for association, usually their *P*-values. An independent replication sample is then analysed by constructing, for each subject, a polygenic score consisting of the weighted sum of its trait-associated alleles, for some subset of top ranking markers. Two related but distinct applications of this score are then possible. Firstly, testing for association between the score and the trait in the replication sample can determine whether associated markers reside within those contributing to the score. Secondly and perhaps more usefully, the polygenic score can be used to predict individual trait values or risks of disease [2], potentially giving a predictor with better discrimination properties than one based on established markers only. Different considerations apply for these two applications, as the size of the replication sample has a direct bearing on the power of association testing, whereas the accuracy of individual predictions depends only on the size of the training sample.

The first successful application of polygenic score analysis to GWAS data was in schizophrenia [3], in which few individual markers were significant and the common disease common variant hypothesis remained in question. It was shown that a large mass, up to half, of all markers in one GWAS could be jointly associated with disease in a second sample, implying a polygenic component to disease risk that justified larger study sizes [4]. Furthermore, markers from schizophrenia GWAS could together be associated with bipolar disorder, and vice versa, establishing a common polygenic basis to those conditions, whereas such cross-prediction was not achieved with clinically distinct conditions such as cardiovascular disease. This common basis has further been exploited to discriminate sub-types of bipolar disorder [5].

Similar results using a large mass of markers have been obtained for other complex traits including multiple sclerosis [6], height [7], cardiovascular risk [8], rheumatoid arthritis [9] and body mass index [10]. In addition, several studies have demonstrated association of a score based on a limited number of top ranking markers [11]–[13]. In some cases, however, the polygenic association is less clear: studies of breast and prostate cancers have been inconclusive, owing in part to technical aspects in analysis but also, potentially, to their sample sizes [14], [15]. An aim of the present work is to determine whether negative results from those studies could be explained by their sample size, or whether a true lack of polygenic effect is the more likely explanation.

Applications of polygenic scores to individual disease prediction have so far been less successful, although proof of concept has been established through simulations [2]. Several studies have shown that a limited number of top ranking markers can discriminate disease cases from unaffected subjects, but the degree of discrimination falls short both of clinical utility and the maximum achievable from genetic data [16]–[18]. The use of a mass of markers across the whole genome has been explored, but to date has not yielded a noticeable improvement in discrimination [14], [19].

Polygenic scores must be estimated from a finite training sample, and their effectiveness for association testing and risk prediction depends on the precision of this estimation as well as the proportion of variation explained by the polygenic score. The role of the sample size has not been thoroughly considered in this context. Several authors have expressed sensitivity and specificity in terms of the genetic variance of a predictor [17], [20]–[22], but they did not distinguish the variance explained by an estimated predictor from that of the true predictor, that is the one that would be estimated from an infinitely large sample. While large samples lead to small sampling variance on individual marker effects, the errors accumulate across multiple markers such that the effect of sampling variation on the polygenic score can be considerable. Wray et al [2] used simulations to study the predictive accuracy of scores estimated from finite case/control studies, but did not obtain an explicit relation between sample size and accuracy. Similarly, the International Schizophrenia Consortium (ISC) [3] used simulations to show empirical relations between sample size and accuracy under several genetic models. Daetwyler et al [23] considered the effect of sampling variation on the correlation between polygenic score and total genetic value. Their results can be adapted to prediction of phenotypes rather than genetic values, and also to other measures of power and accuracy, but their conclusions are limited by an assumption that all the markers have effects and are included in the score.

In this work, statistical properties of polygenic score analyses are derived from a quantitative genetics model as a function of the explained genetic variance and sample sizes in discovery and replication samples. A range of options for constructing the score is considered, including estimation of the score from a different trait to the one predicted, selection of markers according to their *P*-values, and different methods for weighting markers in the score. The power is obtained for testing a polygenic score for association in a replication sample, and the correlation, mean square error, and area under the receiver-operator characteristic curve (AUC) are obtained for a predictor estimated from a finite training sample. These results are used to assess some recent studies and to discuss prospects for the future utility of polygenic score analyses for the prediction of complex traits.

## Results

### Analytic power and accuracy

In the framework considered here, a set of genetic markers is genotyped on an initial training sample and each marker is tested for association to a trait. Effect sizes are estimated for each marker and used to construct a polygenic score for each subject in an independent replication sample. The score is tested for association in the replication sample, in which the tested trait may differ from that in the training sample. The correlation and mean square error between the polygenic score and the tested trait are calculated. If the traits are binary, the AUC is obtained.

More precisely, consider a pair of traits 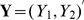 expressed as a linear combination of 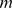 genetic effects and an error term that includes environmental and unmodelled genetic effects:
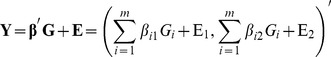
(1)where 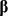 is a 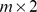 matrix of coefficients, 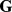 is a 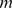-vector of coded genetic markers, and 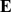 is a pair of random errors that are independent of 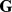. Now suppose that the genetic effects on 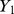 are estimated from a sample of size 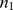 and used to construct a polygenic score to be tested for association to 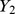 in an independent sample of size 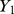. Define the polygenic score to be
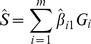
Some important statistical properties of 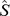 can be expressed in terms of 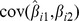 and 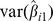, expressions for which are derived in the Methods. The coefficient of determination for the polygenic score on the second trait is
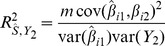
(2)which is the squared correlation between the score and the trait. The prediction mean square error is
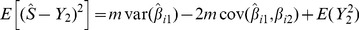
(3)The asymptotic non-centrality parameter of the 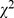 test for association of 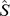 with 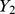 is
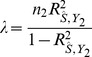
(4)on 1df, and the power of the two-tailed test of association at significance level 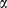 is
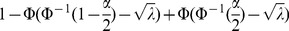
(5)Binary traits are assumed to arise from a liability threshold model [24] leading to calculation of the AUC also in terms of 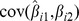 and 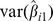, with the expressions given in the Methods. For binary traits the coefficient of determination in equation (2) may be transformed to the liability scale for more satisfactory interpretation [25], the details also given in the Methods.

The expressions for power and accuracy are derived in terms of the parameters listed in Table 1. Estimates of marker effects 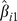 are either obtained from linear regression or set to a signed constant, which corresponds to the common approach of counting risk alleles across markers. A proportion of markers is assumed to have no effect, and markers may be selected by thresholding on their *P*-values.

**Table 1:** Parameters and notation of polygenic model.

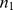	Training sample size
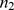	Replication sample size
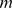	Number of markers in genotyping panel
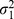	Variance of marker effects in training sample
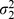	Variance of marker effects in replication sample
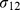	Covariance of marker effects between training and replication samples
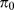	Proportion of markers with no effect in either sample
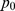	Lower bound on *P*-value in the training sample for a marker to be included in polygenic score
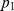	Upper bound on *P*-value in the training sample for a marker to be included in polygenic score
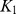	Prevalence of binary trait in training sample
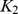	Prevalence of binary trait in replication sample
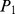	Sampling proportion of cases of binary trait in training sample
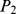	Sampling proportion of cases of binary trait in replication sample

Equation 4 suggests an estimating equation for any parameter of the quantitative model, given the association test between 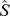 and 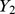. Write 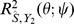 explicitly as a function of some parameter 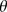 in Table 1, treating all other parameters 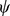 as fixed and known. For example, 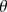 might be the variance of marker effects in the training sample 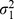, from which 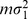 is the explained genetic variance of the marker panel. Alternatively 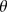 might be the covariance between marker effects in the two samples 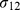, assuming fixed values for the explained variances, and so on. Equation 4 is the squared coefficient of the linear regression of 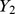 on 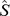, scaled by its sampling variance. The sampling distribution of that coefficient is normal, with mean the square root of equation 4. Therefore applying normal theory an estimator 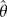 is the solution to the equation
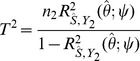
(6)where 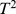 is the observed 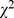 association statistic. An approximate 95% confidence interval for 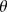 is given by 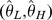 where 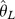 is the solution of
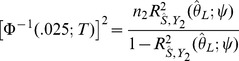
and 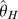 is the solution of
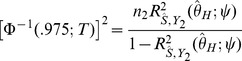



### Published data: Association testing

The ISC was the first to demonstrate the utility of testing polygenic scores [3]. In their main result, odds ratios for 74062 nearly independent SNPs were estimated in 3322 cases and 3587 controls and used to construct a polygenic score that was tested in 2687 cases and 2656 controls of the Molecular Genetics of Schizophrenia study [26]. The score was more strongly associated as higher *P*-value thresholds were used for including SNPs, with the most significant reported association having 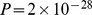 with an inclusion threshold of 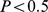.

Assuming a prevalence of 1%, equation 5 gives a power of 80% at nominal significance if the explained genetic variance in liability is 7.2%, rising to 99% if the explained genetic variance is 11.7%. Assuming a heritability of 80% [3] this shows that the test was well powered if the marker panel explains about 10% of the heritability, which seems reasonable. The observed result of 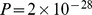 can be used in equation 6 to give an estimated explained genetic variance of 28.7% (95% CI: 23.6%–33.7%), which is 36% of the heritability, assuming that all SNPs have effects that are identical in the two samples. The estimate reduces only to 26.9% if 99% of the SNPs are assumed null. These results are similar to a recent estimate using mixed modelling of the same data [27].

**Figure 1:**
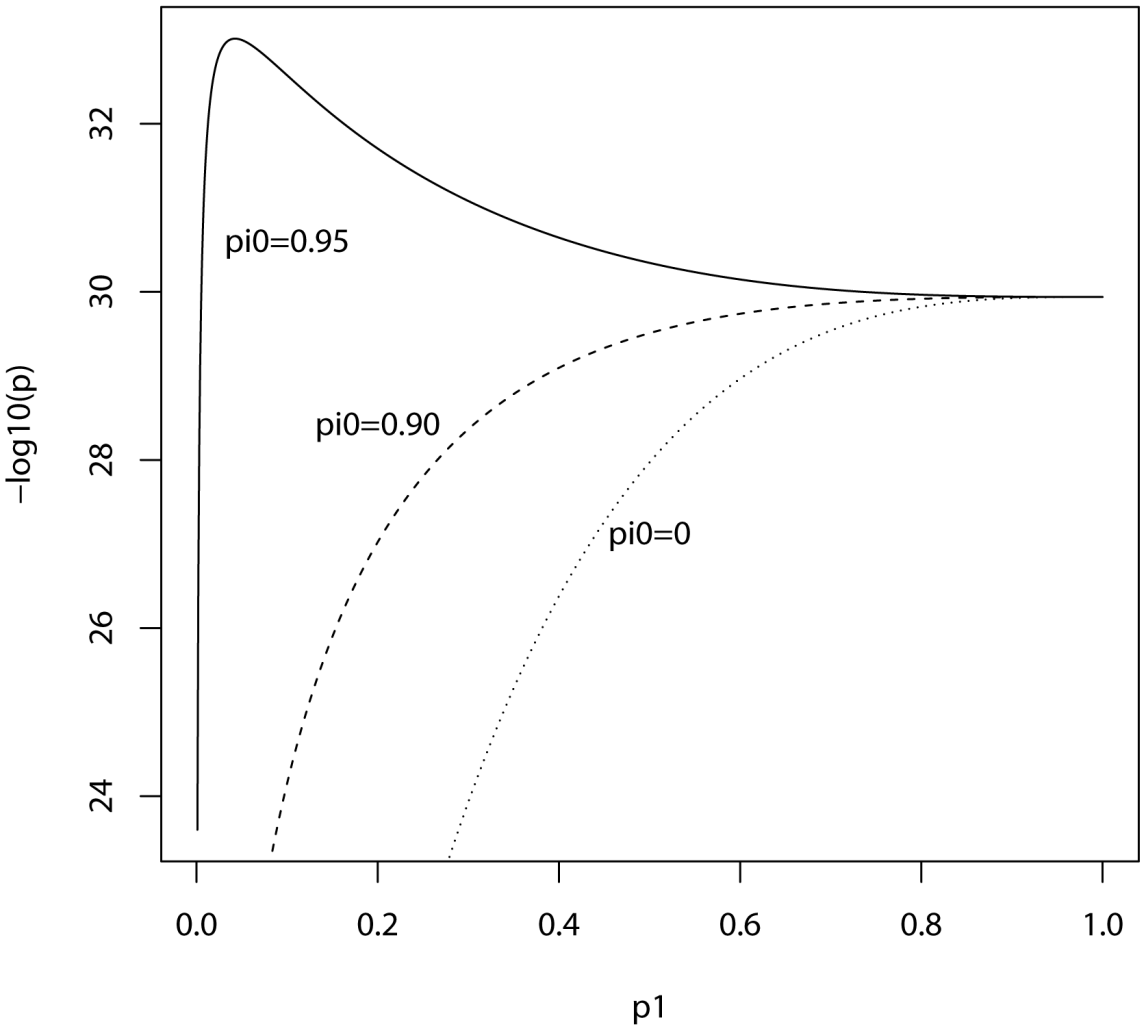
Expected −log_10_(*P*) of linear regression estimate as a function of *P*-value threshold for selecting markers into the polygenic score. Training sample, 3322 cases and 3587 controls; replication sample, 2687 cases and 2656 controls. Marker panel of 74062 independent SNPs. Variance explained by markers, 28.7%. pi0, proportion of markers with no effect on disease.

**Figure 2:**
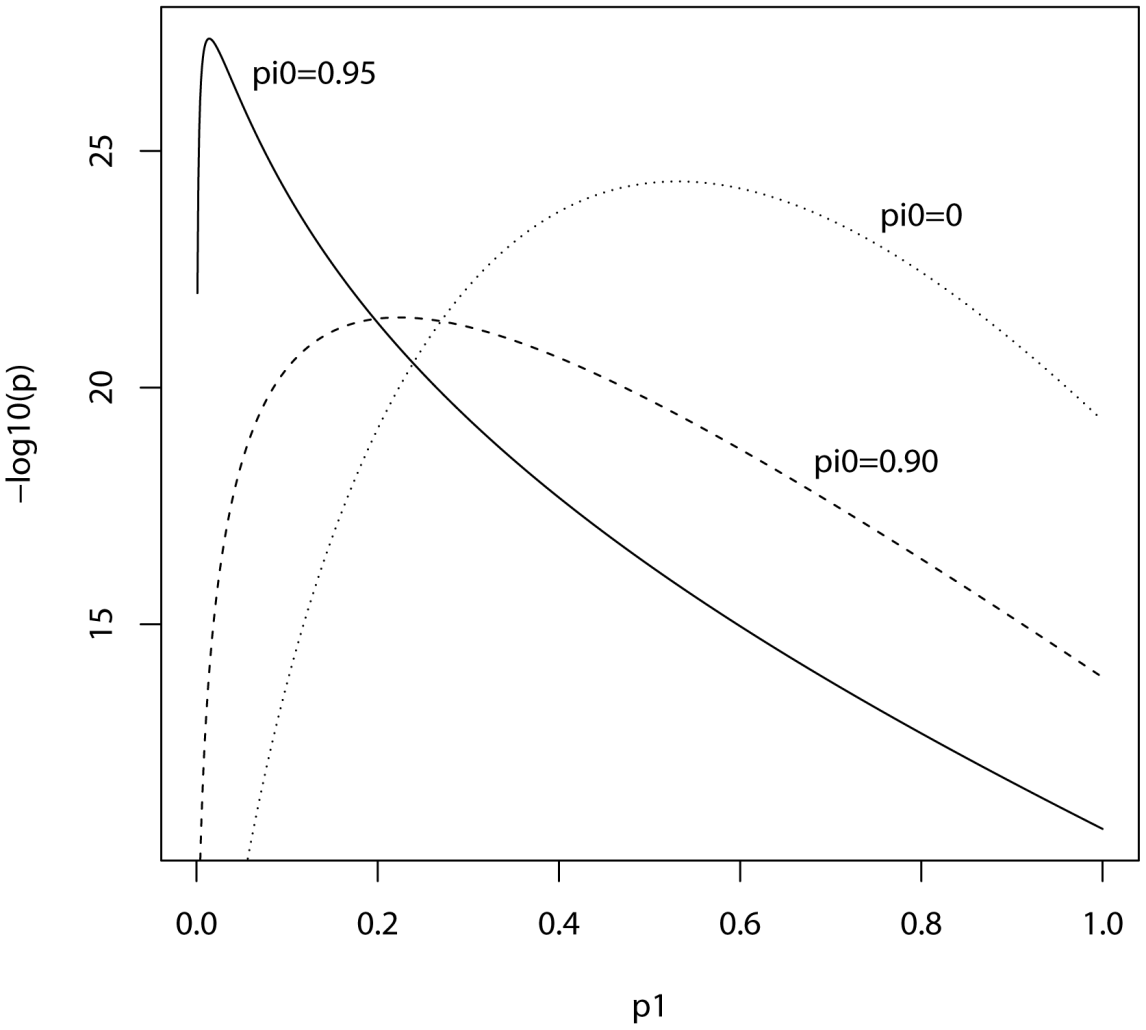
Expected −log_10_(*P*) of allele score estimate as a function of *P*-value threshold for selecting markers into the polygenic score. Training sample, 3322 cases and 3587 controls; replication sample, 2687 cases and 2656 controls. Marker panel of 74062 independent SNPs. Variance explained by markers, 28.7%. pi0, proportion of markers with no effect on disease.

In the ISC report, the *P*-value of the polygenic score decreases as the SNP inclusion threshold increases. This seems to suggest that a large number of associated markers lie within the mass of individually non-significant SNPs. In Figure 1 and Figure 2, the expected *P*-value of the polygenic score is shown as a function of the inclusion threshold, with the explained genetic variance set to its estimated value of 28.7% and other parameters as stated above. The figures show that this trend could be observed when as many as 90% of SNPs have no effects, and for the linear regression estimator the significance of the score continues to improve until the whole marker panel is included. Only for a very high proportion of null SNPs is there an optimal inclusion threshold less than 1. The allele count estimator has an optimum threshold less than 1 for all scenarios, but it is consistently less significant. Thus, in this dataset with high power, decreasing *P*-values are consistent with a range of polygenic models including those with a high proportion of null markers.

In this analysis the replication sample was smaller than the training sample, and we may ask what balance of sample sizes is optimal. Given the total sample size of 3322+2687 = 6009 cases and 3587+2656 = 6243 controls, the non-centrality parameter can be numerically maximised over the proportion of subjects allocated to the training sample. It is found that the optimal split is close to one-half regardless of the proportion of null SNPs or the *P*-value threshold, and the non-centrality parameter is roughly symmetrical around one-half. This suggests that given two samples of different size, it matters little which is chosen for training and which for testing. Furthermore, given an initial sample to be split into training and replication subsets, an obvious rule of thumb is to make an even split. Similar properties are seen under different genetic models (results not shown). Note that these results apply to association testing and not to individual prediction, which is discussed in the next subsection. For association testing there is a balance to be made between the precision of estimating the score in the training subset, and the power of testing the score in the replication subset. For prediction, however, the size of the replication subset does not affect the accuracy, only how precisely it is estimated; thus a larger training subset is more desirable in the prediction context.

The ISC further tested the schizophrenia-derived score against bipolar disorder, to test for a common genetic basis to those conditions. Their strongest result was with the Wellcome Trust Case-Control Consortium (WTCCC) sample of 1829 cases and 2935 controls, obtaining 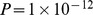 with an inclusion threshold of 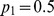. Assume similar heritability for bipolar disorder as for schizophrenia [28] and the same genetic variance explained by the markers, estimated above to be 28.7%. Then using equation 5, the study had 80% power at nominal significance if the correlation is 28% between genetic effects on schizophrenia and bipolar disorder. Using equation 6, the estimated correlation given the observed association statistic is 70.6% (95%CI: 51.3%–89.7%) assuming that all SNPs have effects with explained variance 28.7% in both samples. If 99% of SNPs are assumed null, the estimated correlation reduces to 66.2% (95%CI: 48.1%–84.1%).

The International Multiple Sclerosis Consortium performed a similar exercise using a training sample of 931 cases and 2431 controls, a replication sample of 876 cases and 2077 controls, and a marker panel of 59470 nearly independent SNPs [6]. They also observed decreasing *P*-values for association as more SNPs were included in the score, obtaining 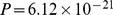 when all SNPs were included. Assuming prevalence of 0.1% this analysis has 80% power at nominal significance for explained genetic variance of 9.4%, and the observed result yields an estimate of 31.5% (95%CI: 24.9%–37.9%) assuming all SNPs have effects.

In applying these ideas to breast and prostate cancers, Machiela et al did not find significant associations of polygenic scores [14]. While this could be explained by the genetic architecture of the diseases, a possible explanation (noted by the authors) is the lower sample size together with the low heritability. Their breast cancer study used a total sample of 2287 subjects, approximately half of which were cases and half controls, which was split into training and testing subsets in a 9∶1 ratio for 10-fold cross-validation. The marker panel consisted of 161,702 nearly independent SNPs. Assuming a prevalence of 3.6% and sibling relative risk of 2.5 [29], this design has only 17% power to detect an association of the polygenic score, even if the markers explain the full heritability. If the sample were split in a 1∶1 ratio, the power would increase to 37%.

Their prostate cancer study had a total of 2277 subjects, approximately half of which were cases, again split in a 9∶1 ratio and a marker panel of 165,508 nearly independent SNPs. Assuming a prevalence of 2.4% and sibling relative risk of 2.8 [29], this design has 19% power if the markers explain the full heritability. If the sample were split in a 1∶1 ratio, the power would be 42%. It is clear that even with the optimistic assumption that the markers explain the full heritability, this study was unlikely to detect an association of the polygenic score for either cancer.

What sample size would have sufficient power to detect association of the polygenic score? For breast cancer the heritability of liability is estimated as 44% [21]. If the marker panel explains half of this heritability, roughly as in the ISC study, then two samples each of 1978 cases and 1978 controls would have 80% power at nominal significance. For prostate cancer the heritability of liability is also 44% and 1766 cases and controls would be required in each sample. For the ISC study, assuming explained genetic variance of 28.7%, 735 cases and controls in each sample are sufficient. Thus it appears that association testing is well powered at current sample sizes if two independent studies are used for training and testing, but less well powered if a single sample is split into two subsets.

As a final example of association testing, this time with a quantitative trait, Simonson et al studied the Framingham Risk Score for cardiovascular disease risk [8]. They also used 10-fold cross-validation of a single sample, giving training samples of 1575 subjects and testing samples of 175 subjects. They used a full set of 250,378 SNPs, which is here assumed to be similar to 100,000 independent SNPs. They first selected SNPs with *P*-values <0.1 into the score, then selected SNPs with 0.1<*P*<0.2, then 0.2<*P*<0.3 and so on, giving ten analyses. Even if the trait is fully heritable and explained by these markers, this analysis has 20% power for the SNPs with *P*<0.1, reducing with each *P*-value interval. For 0.4<*P*<0.5, in which the authors found nominal significance of the score, the power is 6.7%. If all SNPs are included in the score, the power would be 38% if the trait is fully explained by the markers, but under a more conservative model in which the explained genetic variance is 30%, the power is just 8% and increases to 13% under an even split of training and testing samples. Again, splitting a single GWAS sample does not admit high power for testing a polygenic score.

### Published data: Risk prediction

In their study of breast and prostate cancers Machiela et al also calculated the AUC for prediction of disease from the polygenic score. Here it is more important for the training sample to be large, ensuring accurate estimation of the score, justifying the 10-fold cross-validation design. Their AUC did not exceed 53% for breast cancer and 56.4% for prostate cancer. Under the same assumptions as above, the analytic AUC is 53.6% for breast cancer if the markers explain the full genetic variance, or 51.8% if they explain half. However if the sample were infinitely large, the AUCs would be 89% and 79% respectively. For prostate cancer, the analytic AUCs are 54.1% if the markers explain the full genetic variance, and 52% if they explain half; for a large sample they would be 90% and 80%. Thus, the low AUCs observed by Machiela et al are compatible with their study design, but they could be considerably higher if a larger training sample were available.

Evans et al considered prediction for the seven diseases of the WTCCC [19]. Approximately 2000 cases were available for each disease, with a common set of 1480 controls, and a marker panel of all SNPs on the Affymetrix 500K chip after quality control and exclusion of previously known loci. As this is the same chip used in the ISC study, it is assumed here that the panel is equivalent to 74062 independent SNPs. Logistic regression and allele score estimators were both used to construct scores, and a series of *P*-value thresholds from 10^−5^ to 0.8 were considered.

Table 2 compares the results of Evans et al to the analytic AUC for the diseases without strong MHC effects, using *P*<0.8 to select SNPs into the score, as that threshold generally gave the highest AUC. At that threshold, the choice of 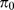 has little bearing on the results unless it is very close to 1, so it is set to 0. Also shown is the maximum AUC possible for each disease, obtained by letting the sample size grow to infinity. Bearing in mind that those authors noticed inflation in AUC for null SNPs, it is again clear that their modest results are compatible with the study design, and more encouraging results might be obtained from a larger sample. The calculations also confirm their observation that the allele count estimator is consistently less accurate than logistic regression; however while the two estimators give similar results at this sample size, more considerable differences emerge in the limit of large samples.

**Table 2:** AUC calculated by Evans et al [19] compared to analytic values when (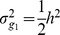) marker panel explains half the heritability, or (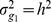) marker panel explains the full heritability.

		Bipolar disorder	Coronary artery disease	Crohn's disease	Hypertension	Type-2 diabetes
	*K*	0.01	0.056	0.001	0.3	0.03
	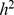	0.69	0.72	0.76	1	0.6
Linear regression	Evans	0.668	0.595	0.614	0.61	0.601
	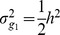	0.570 (0.890)	0.547 (0.843)	0.620 (0.948)	0.539 (0.841)	0.545 (0.832)
	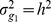	0.638 (0.974)	0.592 (0.948)	0.727 (0.995)	0.577 (0.971)	0.588 (0.934)
Allele count	Evans	0.653	0.599	0.617	0.602	0.589
	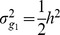	0.561 (0.827)	0.540 (0.780)	0.604 (0.894)	0.533 (0.770)	0.539 (0.772)
	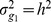	0.620 (0.922)	0.580 (0.880)	0.698 (0.970)	0.567 (0.885)	0.576 (0.868)

*K*, population prevalence and *h* ^2^, heritability of liability taken from Wray et al [21] except for hypertension which is assumed fully heritable for illustration. In parentheses, AUC achieved by an infinite sample.

Several other studies have reported pseudo-*R* ^2^ from the regression of disease on the polygenic score [3], [6], [9]. Although prediction was not emphasised by those studies, they may still be evaluated for that purpose. Recently, Lee et al have argued that, for genetic predictors, *R* ^2^ on the liability scale is a more interpretable measure of accuracy for binary traits [25]. In Table 3, liability *R* ^2^ derived from those reports are compared to analytic values assuming different levels of heritability explained by the markers. The choice of 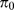 has little bearing on these results so it is set to 0 throughout. The reported values are consistent with the markers explaining around half the heritability, with variation above and below. This is in line with the estimates of explained variance that were reported by those studies, and those estimates also agree well with those obtained using the method proposed here (equation 6). The low reported values of *R* ^2^ do not directly reflect the degree of missing heritability; rather they reflect the effect of sampling variation on the variance explained by an estimated score. Corresponding AUC values are also shown, and it is again clear that the currently modest utility of polygenic scores for discrimination is explained by limited training sample sizes, and much better results are possible through larger samples.

**Table 3:** *R* ^2^ reported for complex diseases compared to analytic values when 
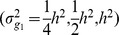
 marker panel explains one quarter, one half or the full heritability.

		Schiz [3]	MS [6]	BrCa [14]	PrCa [14]	RA [9]	Celiac [9]	MI/CAD [9]	T2D [9]
	*K*	.01	.001	.036	.024	.0075	.0075	.056	.03
	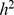	.8	.5	.44	.44	.55	.55	.72	.6
	*π*_0_	0	0	0	0	.97	.98	.98	.96
	Reported 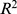	.013	.012	.001	.001	.003	.007	.007	.013
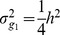	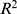	.006 (.2)	.002 (.125)	.0002 (.11)	.0002 (.11)	.001 (.1375)	.0008 (.1375)	.001 (.18)	.003 (.15)
	*AUC*	.56 (.81)	.54 (.81)	.51 (.71)	.51 (.72)	.52 (.52)	.52 (.77)	.52 (.75)	.53 (.75)
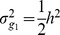	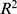	.024 (.4)	.008 (.25)	.0008 (.22)	.0009 (.22)	.006 (.275)	.003 (.275)	.004 (.36)	.010 (.3)
	*AUC*	.62 (.91)	.58 (.90)	.52 (.79)	.52 (.80)	.56 (.87)	.55 (.87)	.54 (.84)	.57 (.94)
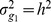	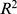	.089 (.8)	.03 (.5)	.003 (.88)	.003 (.44)	.025 (.55)	.013 (.55)	.017 (.72)	.013 (.6)
	*AUC*	.72 (.99)	.66 (.97)	.54 (.89)	.54 (.90)	.62 (.95)	.59 (.96)	.58 (.95)	.63 (.94)
	Reported 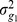	.3	na	na	na	.18	.44	.48	.49
	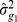	.29	.31	.30	.28	.21	.40	.47	.34

Schiz, schizophrenia. MS, multiple sclerosis. BrCa, breast cancer. PrCa, prostate cancer. RA, rheumatoid arthritis. Celiac, celiac disease. MI/CAD, early-onset myocardial infarction or coronary artery disease. T2D, type-2 diabetes. *K*, population prevalence and *h* ^2^, heritability of liability taken from Visscher et al [1] and Wray et al [21] except for celiac, assumed equal to RA. *π*_0_, proportion of markers assumed to have no effects. Reported *R* ^2^, highest *R* ^2^ reported in cited publication, transformed to the liability scale. In parentheses, values achieved by an infinite training sample. Reported 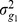, variance explained by markers as estimated in cited publication. 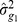, estimated variance explained using method proposed herein.

**Figure 3:**
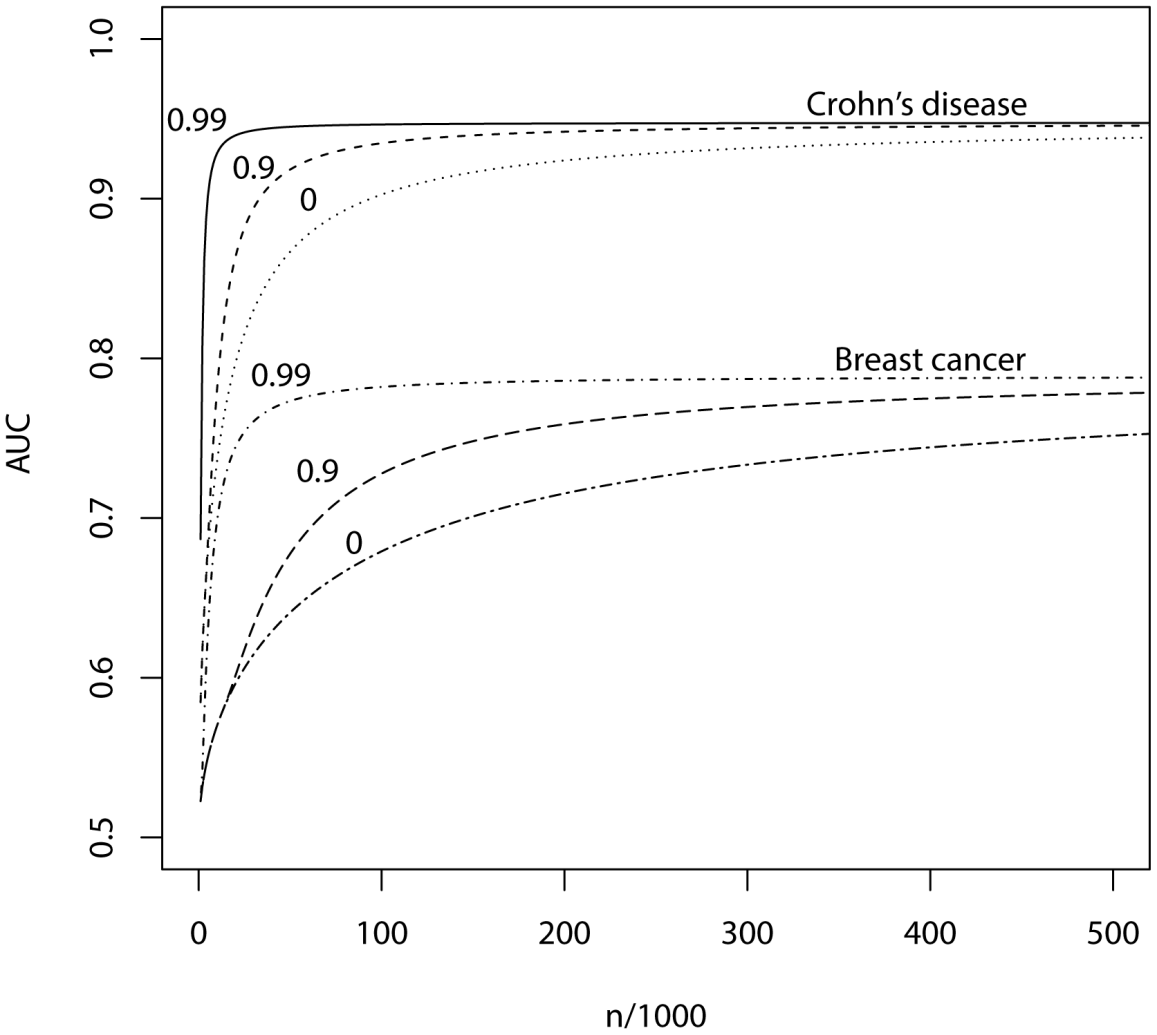
AUC as a function of sample size, using a panel of 100,000 markers that explains half the heritability of liability. *n*, number of cases and of controls in training sample. Heritability of liability, 76% for Crohn's disease. 44% for breast cancer. Line annotations are the proportion of markers with no effect on disease.

What sample size would permit estimation of a score with AUC at a clinical useful level, or otherwise close to its maximum value? The answer depends on 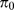, the proportion of null markers in the panel, because if this is high then the individual marker effects will also be high and a low *P*-value threshold will eliminate much sampling error from the estimated score. Figure 3 shows AUC as a function of sample size for Crohn's disease, which has a high heritability of 76%, and breast cancer, which has low heritability of 44% [21], based on a panel of 100,000 independent markers. This is a similar number to current genotyping products, and results are given under a scenario in which the panel explains half the heritability [30]. For each sample size and 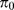, the *P*-value threshold is applied that leads to the highest AUC. An AUC of 0.75 is generally regarded as the minimum useful level for screening subjects already considered at risk, whereas AUC of 0.99 is sufficient for screening the population at large [31]. For these two diseases the latter cannot be achieved from genetic data alone, so Table 4 gives minimum sample sizes for AUC of 0.75 and for 90%, 95% and 99% of the maximum possible AUC given the heritability.

**Table 4:** Numbers of cases and controls (in 1000s of each, rounded up) required to attain a specified AUC using a panel of 100,000 markers that explains half the heritability of liability.

	AUC	π_0_ = 0.99	π_0_ = 0.90	π_0_ = 0.75	π_0_ = 0
Crohn's disease (*h* ^2^ = 0.76, *K* = 0.001, Max = 0.95)	0.75	2 (0.0004)	9 (0.02)	12 (0.5)	12 (1)
	0.855 = 0.9*Max	3 (0.0004)	19 (0.01)	34 (0.06)	42 (1)
	0.9025 = 0.95*Max	6 (0.0004)	35 (0.008)	68 (0.04)	100 (1)
	0.9405 = 0.99*Max	23 (0.0003)	165 (0.004)	349 (0.02)	690 (1)
Breast cancer (*h* ^2^ = 0.44, *K* = 0.036, Max = 0.79)	0.75	23 (0.0004)	157 (0.008)	311 (0.03)	476 (1)
	0.711 = 0.9*Max	12 (0.0005)	77 (0.01)	144 (0.05)	183 (1)
	0.7125 = 0.95*Max	23 (0.0005)	159 (0.01)	315 (0.05)	484 (1)
	0.7821 = 0.99*Max	100 (0.00024)	755 (0.00389)	1610 (0.0147)	3281 (1)

π_0_, proportion of SNPs having no effect on disease. Max, maximum AUC achievable given the genetic variance of the marker panel. In parentheses, *P*-value threshold that maximises the AUC.

The most favourable condition shown is 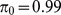, that is there are 1000 markers with effects on disease. Figure 3 and Table 4 show that a few thousand cases and controls could yield a clinically useful AUC, but under most conditions several tens of thousands are needed. Under less favourable conditions – low heritability, low proportion of null markers – several hundred thousand cases and controls are needed to obtain an AUC within 10% of the achievable level, and even an AUC of 0.75 requires some tens of thousands of subjects. In the worst case the order of magnitude is of the millions.

**Figure 4:**
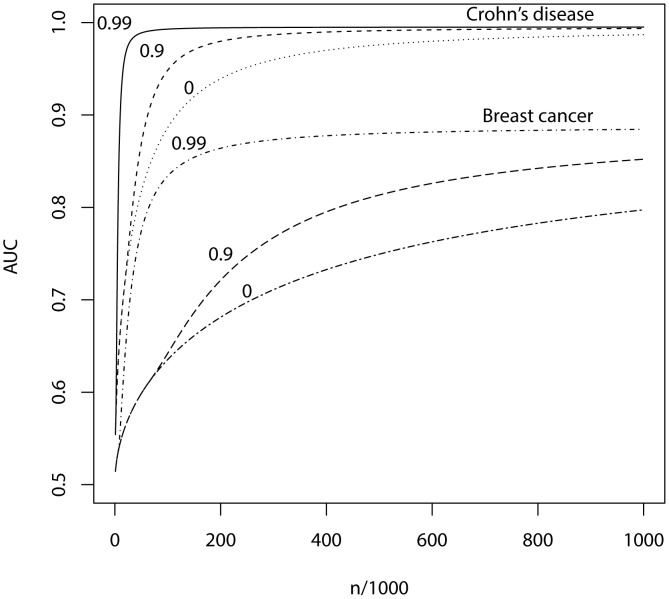
AUC as a function of sample size, using a panel of 1,000,000 markers that explains the full heritability. *n*, number of cases and of controls in training sample. Heritability of liability, 76% for Crohn's disease. 44% for breast cancer. Line annotations are the proportion of markers with no effect on disease.

Whole genome genotyping is now becoming feasible, under which the entire narrow-sense heritability would be represented. Assuming this is equivalent to about one million independent common SNPs [32], the required sample sizes are shown in Figure 4 and Table 5. Again, unless the heritability is explained by about 1000 markers, several tens to hundreds of thousands of subjects are needed to obtain a clinically useful AUC; for the genetic predictor to approach its potential, the order of magnitude is of the millions. The sample sizes to achieve AUC of 0.75 are larger than for 100,000 SNPs explaining half the heritability, but the latter scenario cannot achieve AUC of 0.99, so the clinical context can influence the choice of marker panel used to derive the predictor. It is clear that at current sample sizes, polygenic scores are only going to approach useful levels of discrimination if the marker panels include a high proportion of associated loci and the number of such loci is relatively small. Furthermore, for highly polygenic conditions the sample sizes needed to approach this potential are an order of magnitude higher than are currently available.

**Table 5:** Numbers of cases and controls (in 1000s of each, rounded up) required to attain a specified AUC using a panel of 1,000,000 markers that explains the full heritability.

	AUC	π_0_ = 0.999	π_0_ = 0.99	π_0_ = 0.90	π_0_ = 0.75	π_0_ = 0
Crohn's disease (*h* ^2^ = 0.76, *K* = 0.001, Max = 1.00)	0.75	1 (0.00007)	5 (0.0004)	25 (0.08)	27 (1)	27 (1)
	0.9 = 0.9*Max	2 (0.00007)	10 (0.0004)	62 (0.01)	107 (0.1)	117 (1)
	0.95 = 0.95*Max	3 (0.00007)	16 (0.0005)	103 (0.01)	190 (0.05)	243 (1)
	0.99 = 0.99*Max	8 (0.00007)	58 (0.0003)	413 (0.006)	847 (0.02)	1487 (1)
Breast cancer (*h* ^2^ = 0.44, *K* = 0.036, Max = 0.89)	0.75	6 (0.00007)	41 (0.0004)	256 (0.01)	448 (0.09)	505 (1)
	0.801 = 0.9*Max	9 (0.00007)	65 (0.0005)	428 (0.009)	806 (0.05)	1062 (1)
	0.8455 = 0.95*Max	17 (0.00007)	124 (0.0004)	857 (0.007)	1702 (0.03)	2656 (1)
	0.8811 = 0.99*Max	77 (0.00007)	566 (0.0002)	4305 (0.004)	9223 (0.01)	19191 (1)

π_0_, proportion of SNPs having no effect on disease. Max, maximum AUC achievable given the genetic variance of the marker panel. In parentheses, *P*-value threshold that maximises the AUC.

Finally, Table 6 gives similar calculations for the correlation between predicted and observed quantitative traits with high (*h* ^2^ = 0.8) and moderate (*h* ^2^ = 0.4) heritability. The prospects here appear more challenging in terms of the sample sizes needed to approach the achievable correlation. For example, height has heritability of about 0.8, and the number of associated variants is known to be at least in the hundreds [7]. In the most optimistic scenario shown, 31,000 subjects would be required to derive a predictor with correlation 0.8 with the true height. In fact these sample sizes are now being approached by collaborative studies, and this result confirms that this is necessary for accurate prediction of quantitative traits in addition to the primary goal of identifying individually associated markers.

**Table 6:** Numbers of subjects (in 1000s, rounded up) required to attain a specified correlation with a normal trait using a panel of 1,000,000 markers that explains the full heritability.

	Correlation	π_0_ = 0.999	π_0_ = 0.99	π_0_ = 0.90	π_0_ = 0.75	π_0_ = 0
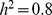 (Max = 0.894)	0.8046 = 0.9*Max	31 (0.00007)	227 (0.0004)	1601 (0.007)	3231 (0.03)	5329 (1)
	0.8493 = 0.95*Max	55 (0.00007)	411 (0.0003)	3004 (0.005)	6250 (0.02)	11571 (1)
	0.88506 = 0.99*Max	213 (0.00007)	1546 (0.0002)	12171 (0.003)	26724 (0.01)	61565 (1)
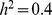 (Max = 0.632)	0.5688 = 0.9*Max	61 (0.00007)	453 (0.0004)	3201 (0.007)	6461 (0.03)	10658 (1)
	0.6004 = 0.95*Max	109 (0.00007)	821 (0.0003)	6007 (0.005)	12500 (0.02)	23141 (1)
	0.62568 = 0.99*Max	426 (0.00007)	3092 (0.0002)	24341 (0.003)	53448 (0.01)	123128 (1)

π_0_, proportion of SNPs having no effect on the trait. Max, maximum correlation achievable given the genetic variance of the marker panel. In parentheses, *P*-value threshold that maximises the correlation.

## Discussion

To date polygenic score analyses have been performed opportunistically. The results provided here allow a more informed appraisal of these analyses, characterisation of the statistical properties of the methods, and insights into the future prospects of polygenic modelling. R code to compute the formulae in this paper is available from the author (sites.google.com/site/fdudbridge/software/).

Current sample sizes are clearly adequate for testing association of a polygenic score in a replication sample, as long as full size samples are used for both training and testing. This is already apparent from the extraordinary significance levels reported in the seminal studies [3], [6], but here it is shown that those results are compatible with realistic genetic models and are not necessarily explained by analytic biases that accumulate across SNPs. This had been previously shown by the ISC study, which simulated plausible genetic models and showed that they led to similar results to those observed in the data [3]; here, the result is shown directly for the common quantitative model, without recourse to simulations. Studies that split a single sample into cross-validation subsets have been less successful [8], [14], but here it is shown that this could be explained by their limited sample sizes, and more encouraging results for the same traits could be obtained with modestly increased samples.

When a sample is to be split into two subsets, a roughly even split yields the greatest power for testing association of the score. However, for predicting individual trait values it is more important for the training set to be large, and standard procedures such as 10-fold cross-validation remain preferable. If both testing and prediction are intended from a single sample, a pragmatic approach is to ensure adequate power by allocating about 2000 cases and 2000 controls to the replication sample, providing this is less than half the total, and then to ensure high predictive accuracy by allocating the remainder to the training sample.

The outlook for disease and trait prediction is more challenging. To date the severe shortfall in the accuracy of genetic predictors has generally been ascribed to incomplete coverage of marker panels or failure to identify sufficiently many associated markers. Here, however, no criteria for declaring individual significance are imposed, but neither does the calculation force the predictor to include markers that contribute no information. Under this pragmatic approach it results that tens of thousands of subjects, at least, are needed to derive predictors that are clinically useful. Furthermore, previous results on the potential accuracy of genetic prediction [17], [20]–[22] only become relevant at very large sample sizes. Such numbers are now coming within reach of national biobank projects and international consortia, so the emergence of useful genetic predictors may not be too far off, although such large samples create issues of effect heterogeneity that are not addressed here. Recent estimates of the proportion of markers having effects also suggest that the more optimistic scenarios shown in Table 4, Table 5, Table 6 may apply [9], [33]. Although the focus here is on AUC, various other measures of predictive accuracy are possible and can be computed within the same framework [24], [25]. The expressions given here could be adapted to other measures without much difficulty.

For some diseases, fairly high AUC has already been observed [17], [19]. This does not conflict with the present work but reflects the presence of major gene effects, usually in the MHC, which depart from the quantitative model treated here. Similarly, some diseases have non-genetic risk factors that already admit clinically useful predictors. There the more relevant issue is the extent to which genetics improves established models [34]. Again the focus has tended to lie on identifying specific markers to improve prediction, rather than the sample size needed to accurately estimate their combined effects. The approach taken here could easily be extended to accommodate additional fixed effects.

A fairly general construction of the polygenic score has been described, including weighted and unweighted methods from single marker analysis, and shrinkage methods used in multivariate analysis. There is little to choose between these estimators in terms of power, correlation or AUC, but the unweighted estimator will perform relatively worse as sample size increases since its sampling error does not reduce to zero. Shrinkage estimation leads to reduced mean square error for prediction and has some other advantages [35], [36], but in the main applications for polygenic scores to date, namely association testing and AUC, it does not improve over the linear regression estimate.

However, some ideal conditions have been assumed including independence of markers and of study subjects. In reality markers will be in linkage disequilibrium and the approximation by an effective number of independent tests is heuristic. Similarly, subjects will be related, if distantly. Results from real data may depart from those presented here if proper account is taken of relationships between subjects. In particular, shrinkage estimation is likely to improve power and correlation, as well as mean square error, by analysing all markers simultaneously rather than each one marginally [37].

The assumption that effects are normally distributed is necessary when markers are selected by their *P*-values but not otherwise. Similarly, allowing a proportion of markers to have no effect only makes a difference when selecting markers by *P*-values. Thus the present results are relevant even if one does not entirely accept the polygenic model proposed. The normal distribution simplifies some calculations, but various heavy-tailed distributions have also been proposed for GWAS data [38], [39] and would lead to improved prediction if such models held in truth. Furthermore the assumption of normality applies to effects on the standardised genotype scale, but there are plausible models for effect sizes as a function of allele frequency, leading to non-normal effects on the standardised scale. This may particularly affect the results for shrinkage estimation when the degree of shrinkage varies for markers with different allele frequencies. The numerical results presented are therefore not definitive but should be taken a guide to the likely magnitude of results in specific applications.

A novel approach to estimating parameters of the polygenic model has been proposed, showing promise for inferring the explained genetic variance and/or proportion of null markers. The method yields estimates that are similar to those obtained by existing approaches [1], [37]. A similar approach to estimation has been developed by Stahl et al [9], based on simulating GWAS data from proposed models, and using rejection sampling to construct posterior distributions of their parameters. Apart from the accommodation of prior distributions (which were uninformative), this is essentially the same approach as used here except that whole genome simulation is used to obtain a sampling distribution. The analytic results provided here should allow this approach to be implemented more efficiently, and this will be attempted in future work.

The *P*-value thresholds that maximise the power and AUC are more permissive than the usual thresholds for individual markers. This means that polygenic analyses can be powerful while still including many non-significant markers, so they will continue to be useful as long as individually associated markers remain to be discovered. Although larger samples are needed for useful risk prediction, polygenic scores have an ongoing current role in assessing the variance explained by marker panels and the genetic correlation between related traits and populations.

## Methods

### Quantitative model

Recall equation 1 in which a pair of traits 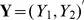 is expressed as a linear combination of 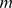 genetic effects and an error term that includes environmental and unmodelled genetic effects:
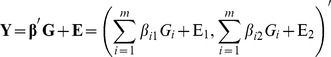
where 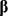 is a 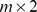 matrix of coefficients, 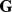 is a 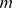-vector of coded genetic markers, and 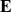 is a pair of random errors that are independent of 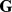. Assume that the markers 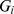 are independent and standardised. In the usual case of single nucleotide polymorphism (SNP) genotypes under Hardy-Weinberg Equilibrium, 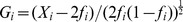 where 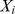 is the number of minor alleles and 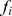 is the minor allele frequency at SNP 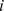. The genetic effects 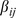 are regarded as fixed across samples but random over 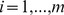 with 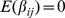, 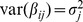 and 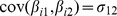. Then the variance-covariance matrix of 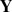 is written as
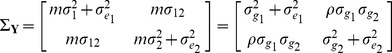
For continuous traits, assume without loss of generality that 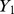 and 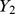 are standardised so that 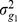 and 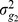 are the proportions of variation of each trait explained by 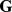. These quantities will be called the explained genetic variances, and are bounded above by the heritabilities.

The genetic effects on 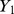 are estimated from a sample of size 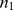 and used to construct a polygenic score to be tested for association to 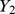 in an independent sample of size 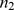. Define the polygenic score to be
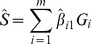
Clearly 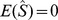. Furthermore if 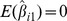 then
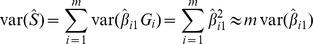


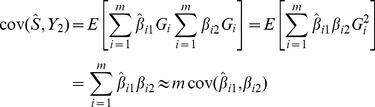
(7)These expressions are equalities in the limit of large 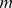 but are approximations for a finite number of markers because the true effects 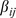 are a sample from their random effects distribution.

Equations 2–6 in Results follow immediately, in which the key quantities are 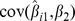 and 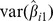. They in turn depend upon the form of the estimator 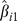, for which three alternatives are now discussed.

### Linear regression

A natural estimate of 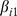 is the least squares estimate from the univariate linear regression of 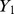 on 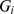. Then 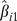 is asymptotically normally distributed with sampling mean 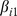 and variance 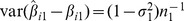 since 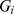 is standardised by definition. Assuming that genetic effects are small, it is henceforth conservatively taken that 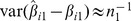 as previously suggested by Daetwyler et al [23]. The total variance of this estimator over markers and samples is 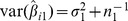, and its correlation with the effects on 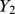 is
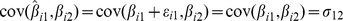
where 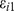 are the sampling errors. Immediate power and accuracy calculations are then available by substituting 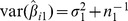 and 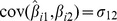 into equations 2–5. When 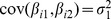, as when the same trait is considered in both samples, equation 2 gives the formula previously derived by Daetwyler et al [23], modified to allow for prediction of the phenotype rather than the genetic value. In the present notation,
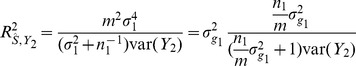
corresponds to equation 1 of those authors, with the additional factor 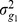 being the genetic variance of the phenotype. This shows that the key determinants of the predictive accuracy are the variance explained by the markers and the ratio of the sample size to the number of markers.

Now suppose markers are only selected into the polygenic score if they have two-tailed *P*-values between thresholds 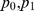 where 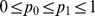. Asymptotically the equivalent constraint for 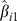 is obtained from the Wald statistic as
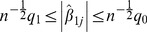
(8)where 
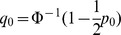
, 
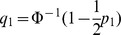
.

Suppose further that a proportion 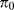 of the 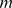 markers have no effect on 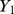 (*ie.* 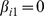), and the remaining markers have effects drawn from 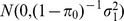. Then among the null markers the variance of 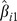, conditional on selection into the polygenic score, is obtained from properties of the truncated normal distribution as [40]

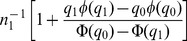
Similarly, among the non-null markers the variance of 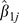, conditional on selection into the polygenic score, is
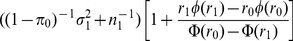
(9)where 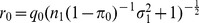, 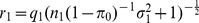. The probability that a null marker is selected into the polygenic score is 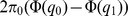 and the corresponding probability for a non-null marker is 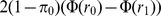. Therefore the total variance of 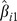 is
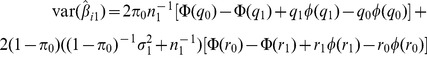
(10)Note that when 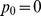 and 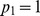, that is all markers are included in the score, then equation 10 reverts to 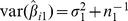, which is invariant to the proportion of null markers 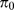 and does not assume a normal distribution for the non-null effects [23].

To obtain 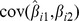 allowing for selection of markers, note that the regression of 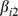 on 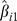 has the same coefficient regardless of selection on 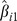. For non-null markers this coefficient is
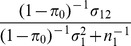
and the covariance is this coefficient times the conditional variance of 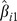 given in equation 9. For null markers the covariance is zero, so the total covariance is
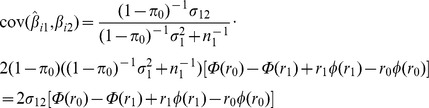
This expression is substituted into equations 2–5 together with the variance in equation 10 to obtain the power and accuracy of the polygenic score when markers are selected into the score based on their *P*-values.

### Shrinkage estimation

It is well known that estimation and prediction for multivariate models can be improved, in terms of mean squared error, by assuming that their effects come from a common underlying distribution. A common approach in quantitative genetics is to fit a mixed model in which genetic markers have random effects for which best linear unbiased predictors (BLUPs) are obtained [35]. This is one of several closely related formulations of multilevel models [36]. As these approaches tend to give similar results when the number of markers is large, a basic Bayesian estimation scheme is outlined here and will be assumed to give typical results for a shrinkage estimator.

Suppose 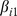 has the prior distribution 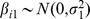, and let the “data” consist of the univariate linear regression estimates, 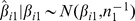. Then the posterior for 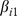 given 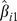 is also normal, 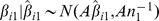 where 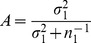 [41]. A natural estimator for 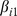 is therefore the posterior mean 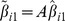 for which 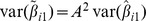
and 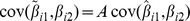. Since all effects are shrunk by the same factor 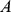 it follows that this approach leads to the same power and correlation as the linear regression estimator 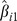, but the mean square error is reduced to 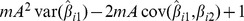.

### Allele count

A currently common approach is to construct the polygenic score by summing the number of trait-increasing alleles across selected markers, without considering their effect sizes other than to identify the direction of association at each marker. This may be called an unweighted score, in contrast to the above approaches that estimate weights for each marker. The unweighted score may be more robust against errors in estimating the effect sizes arising from limited sample size, population heterogeneity, “winner's curse” bias, and confounding by population structure. Here a related approach is considered in which all markers are given the same absolute effect size on the standardised genotype scale. This is equivalent to the allele counting approach when all markers have the same allele frequency. When allele frequencies are heterogeneous, allele counting assumes that all markers have the same effect on the trait, whereas the present approach assumes that all markers contribute the same proportion of variance to the trait. Both models can be criticised but the present approach will allow the comparison of weighted to unweighted scores without considering the distribution of allele frequencies or their relation to the effect sizes.

The polygenic score is now calculated as
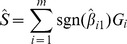
where 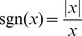 and 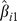 is the linear regression estimate as before. Clearly 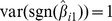. The covariance 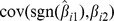 is obtained by integrating over the distribution of 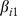. Allow again for selection of markers by their *P*-values as in equation 8 and denote the selection event by 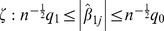. Then using the symmetry of the distribution of 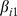 the required covariance is
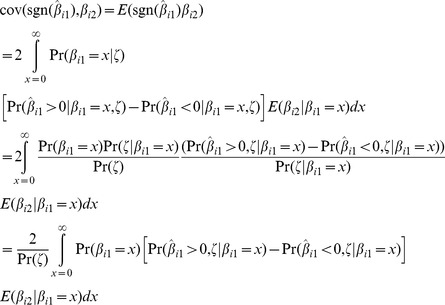
The probabilities in this expression are as follows. The selection probability is again
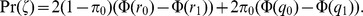
The probability density for nonzero 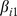 is
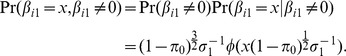
Given some value of 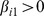 the probability that its estimator is also positive, and the marker is selected into the score, is
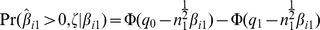
Similarly given 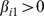 the probability that its estimator is negative, and the marker is selected, is
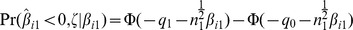
Finally the conditional mean of 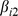 given 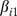 is given by properties of the bivariate normal distribution as 
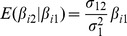
. The integral can be evaluated numerically, yielding values for power and accuracy from equations 2–5.

### Binary traits

The forgoing is based on linear regression, which is the usual approach for quantitative traits. For binary traits the standard analysis is logistic regression, used both for estimating the coefficients 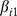 in the polygenic score and for testing the association of the score in a replication sample. For small effects the log-odds are approximately linear in the predictors, so we may continue to work in a linear regression framework for estimating power and accuracy. That is, the binary trait is coded as 0/1 and treated as the response in ordinary linear regression. The variance of 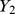 in equation 2 is now the binomial variance 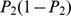 where 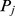 is the proportion of study subjects with 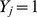. In a prospective sample, 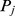 is the population proportion of the trait, whereas in a case/control sample (to be discussed further below), it is the sampling proportion of cases.

The binary traits are now assumed to arise from a liability threshold model, under which all individuals have an underlying normally distributed trait, called the liability, and all those whose liability exceeds a fixed threshold will exhibit the trait. Although the liability is not directly observed, this model has several advantages for modelling polygenic effects, including independence of the genetic effects from the trait prevalence, and an elegant linear transformation between effects on liability to corresponding effects on the observed (0/1) trait. This model has recently been elucidated by several authors for studying the quantitative genetics of binary traits in humans, and the reader is referred to their papers for more detailed discussion [21], [24], [30].

Assuming the marginal liabilities 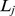 are distributed as a standard normal, the threshold for exhibiting trait 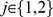 is 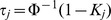 where 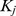 is the population prevalence. The genetic effects 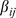 are now taken to act on liability, and for small effects a linear transformation to the corresponding effect on the observed trait may be obtained as [30]

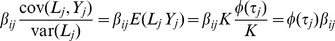
(11)Given the genetic variance-covariance matrix 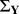 on the liability scale, the statistical properties of the polygenic score may now be calculated as before, but substituting 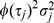 for 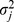 and 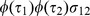 for 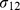 throughout, and using 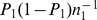 as the sampling variance of 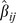.

Sensitivity and specificity are often of interest in the prediction of binary traits. In particular, the accuracy of a predictor can be assessed by the AUC constructed as follows. Subjects are classified such that those with a polygenic score above a fixed threshold are predicted to have the trait, those below the threshold to not have it. Sensitivity is the proportion of subjects with the trait who are correctly predicted as such, and specificity the proportion of subjects without the trait correctly predicted as such. Each possible threshold leads to a value of sensitivity and specificity, defining the receiver operator characteristic curve by plotting sensitivity against 1-specificity. The AUC can be defined as the probability that a pair of subjects, one with the trait and one without, is correctly classified by the predictor. Because the central limit theorem implies that the polygenic score is normally distributed, the expected AUC can be calculated as [21]

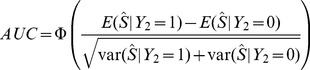
(12)In this expression, 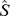 is formed from effects on 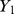 estimated on the observed scale whereas the conditional means and variances are conveniently calculated on the liability scale for 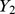. There is a linear transformation between effects on 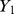 and those on 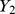, defined by their bivariate normal distribution, and equation 11 gives another linear transformation between effects on 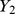 and those on 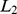. Equation 12 may therefore be equivalently written in terms of effects on 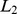 with the corresponding score denoted 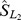. The conditional means and variances are functions of the variance in 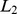 explained by 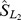 [21], [40], which is 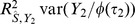, giving
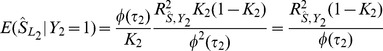
and
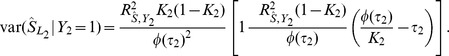
Similarly
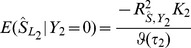
and
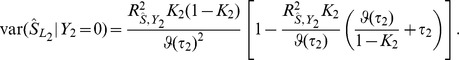



### Case/control studies

In case/control studies the increased ascertainment of cases leads to departure from the normal distribution of liability assumed in the previous subsection. To overcome this problem, it is again assumed that there is a linear transformation from an effect 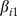 on liability to one on the observed trait in which the 0/1 response denotes ascertained case status.

When there is no selection on 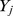 or 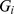 the regression of 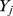 on 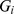 has coefficient 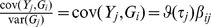 from equation 11. The converse regression of 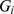 on 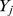 has coefficient 
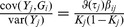
. The latter will also apply when there is ascertainment on 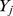, but the regression of 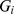 on ascertained 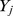 can also be written as 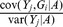, where 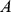 denotes ascertainment, so that
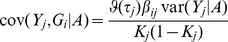
The desired quantity is the coefficient for the regression of ascertained 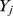 on 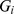 which is thus
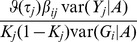
In general the variance of genetic markers 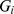 will differ from 1 under ascertainment but it will henceforth be assumed that its expectation over markers is approximately 1. A heuristic justification for this assumption is given in the Text S1. Based on this assumption, an effect 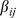 on liability is transformed by the factor
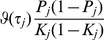
(13)to the observed case/control scale. Similarly to before, given the genetic variance-covariance matrix 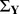 on the liability scale, the properties of the polygenic score can be calculated on the observed scale, substituting 
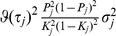
 for 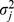 and 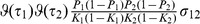 for 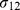 throughout, and using 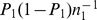 as the sampling variance of 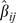.

To obtain the AUC, the same approach as before is used, but now using
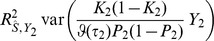
as the variance in 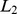 explained by 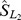. Therefore,
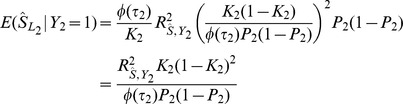
and
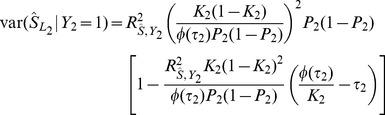
Similarly
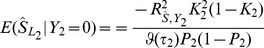
and
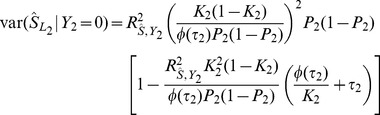
The transformation from liability to observed scales differs from that of Lee et al [30], which is for the total genetic liability (their equation 19). Here the interest is in the individual marker effects on the observed scale, because they are what are estimated when constructing the polygenic score.

### Liability *R* ^2^

The derived expressions involve 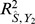 which is the coefficient of determination on the observed scale. Lee et al have argued that, for a genetic predictor, *R* ^2^ on the liability scale is more interpretable for binary traits as it is invariant to the population prevalence and sampling ratio [25]. An approximate transformation to the liability scale is obtained by transforming the genetic effects using equation 13 and rescaling the trait variance from the binomial variance on the observed scale to the unit variance on the liability scale. Therefore,
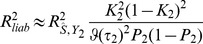



### Log-risk model

An alternative to the liability threshold model is the log-risk model for binary traits, which is equivalent to the logistic model in the limit of low prevalence. Here the polygenic score estimates the log risk of disease, which is assumed to be normally distributed in the population with mean 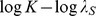 and variance 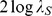, where 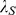 is the sibling relative recurrence risk [17], [20]. Under this model the log risk has the same variance in cases and controls, but the mean log risk among cases is increased by that same variance, becoming 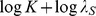. This model allows a simpler calculation of AUC for rare disease, which is given here but not pursued further.

Given 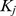 and 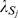 and denoting log-risk of trait 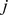 by 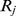, the transformation from log-risk to observed scales is
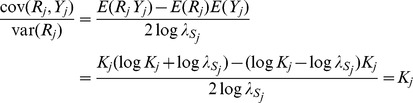
with the same adjustment for case/control ascertainment (equation 13). The difference in polygenic scores between cases and controls is the variance of the score,
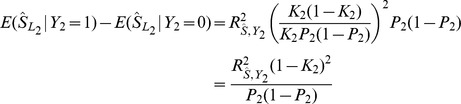
Since the polygenic score has the same variance in cases and controls, equation 12 gives the AUC as
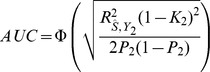



### Simulations

The derived expressions were compared to simulations in which the major assumptions were examined under realistic scenarios. These assumptions include a large number of markers with effects, for equality in equation 7, and small genetic effects, so that effects on the liability scale are approximately linear. In case/control designs the disease prevalence is assumed to be not too small, so that the variance of the ascertained genotypes remains near 1 as assumed in equation 13 and Text S1. Effects are assumed to be normally distributed on the standardised genotype scale. Sample sizes are assumed large so that estimates of genetic effects are normally distributed.

A baseline scenario was defined to reflect that seen in recent studies, as follows. Two normally distributed traits were simulated with explained genetic variances 0.4 and 0.3 and correlation of genetic effects of 0.65. Genotypes from 100,000 independent SNPs were simulated, with minor allele frequencies uniformly distributed on (0.01, 0.5). This reflects current marker panels that directly explain about half the heritability [1]. The proportion of null SNPs was 0.95 or 0.99 [9], with the same SNPs having effects for both traits. Their effect sizes were drawn from the bivariate normal distribution such that the desired variances and covariance were attained. The traits were then generated from the quantitative model in equation 1.

The polygenic score was estimated using the first trait in a sample of 4000 unrelated subjects. The score was constructed using *P*-value thresholds of 0.1 and 0.001 for π_0_ = 0.95 and 0.99 respectively; these thresholds yielded the highest *R* ^2^ and AUC values. The score was then tested for association with the second trait in an independent sample of 4000 subjects. The correlation and mean square error between the score and the second trait were also estimated in the second sample. The association tests were used in equation 6 to estimate the explained genetic variances in the first and second samples in turn, and then the covariance between effects in the two samples, each time keeping other parameters fixed to their simulation values.

Table S1 shows estimates from 1000 simulations compared to the analytic values, for the three estimators discussed. Mean square error for the allele count estimator is not meaningful without further scaling of the polygenic score, which is a further problem not of present interest. All simulations agree well with the analytic results. Because the variances and covariance are bounded in (0,1), their median estimates are shown with the coverage, rather than their means. The proposed estimating equations are seen to be accurate, but the confidence intervals are anti-conservative when the number of markers with effects is low, here 1000. This is because the realised variance and covariance in equation 7 depart from their large *m* expectation, with resulting over-dispersion in the estimating equation (left hand side of equation 6). However when the number of markers with effects is 5000, the correct coverage is attained.

The traits were then treated as liabilities for binary diseases with prevalence 0.2. Disease status was simulated prospectively, as in a cohort study. The polygenic score was estimated and tested using both linear and logistic regression. Table S2 shows estimates of power and AUC compared to the analytic values. Results for the shrinkage estimator are identical to the regression estimator and are not shown. All simulations agree well with the analytic results, and the proposed estimating equations are accurate. The results for logistic regression agree well with those for linear regression, justifying the use of the latter to derive the analytic results.

Then, a case/control design was simulated in which the disease prevalence was now 0.001. The same total sample sizes were used but included equal numbers of cases and controls. A computationally efficient approach to this simulation is described in Text S2. The results are given in Table S3. Again all simulations are seen to agree with the analytic values, but when the number of markers with effects is low, there is a downward bias in the parameter estimates and the confidence intervals of the parameter estimates are anti-conservative. Again the logistic regression results agree well with those for linear regression. Taking Tables S1, S2, S3 together, the analytic methods are accurate for the strongest effects likely to be seen in current studies, but when the number of SNPs with effects is about 1000, there is downward bias in the effect estimates and under-coverage of the confidence intervals, the degree of which appears to vary with the strength of the association.

To assess robustness to normality of the marker effects, the simulations were repeated with the effects drawn from Laplace distributions and then rescaled to give the same explained variance and correlation as before. Instead of π_0_ = 0.95 and P<0.1, simulations with π_0_ = 0 and P<1 were performed to verify that this situation does not assume normality. The results in Table S4, Table S5 and Table S6 confirm this to be the case, whereas when π_0_ = 0.99 and P<0.001 the analytic expressions tend to underestimate the power and accuracy. This is due to the heavier tails of the Laplace distribution compared to the normal, and quantitatively different results would be seen for different generating models. Again, bias and under-coverage is seen when there are 1000 markers with effects.

## Supporting Information

Table S1Simulations of quantitative traits compared to analytic results. Analytic values are in parentheses. Genotypes for 100,000 SNPs were simulated in 4000 subjects in each of two samples. Minor allele frequencies were drawn from Unif(0.01,0.5). Effect sizes in the two samples were drawn from the bivariate normal distribution with marginal variances 0.4, 0.3 and correlation 0.65. π_0_, proportion of SNPs having no effect on traits. *P*, *P*-value for including SNP in the polygenic score. NCP, non-centrality parameter. Power computed at α = 0.05. MSE, mean square error. 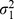, 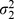, 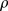, median estimates of model parameters, with coverage of 95%CI in brackets.(DOCX)

Table S2Simulations of binary traits in prospective samples compared to analytic results. Analytic values are in parentheses. Genotypes for 100,000 SNPs were simulated in 4000 subjects in each of two samples. Minor allele frequencies were drawn from Unif(0.01,0.5). Effect sizes on liability were drawn from the bivariate normal distribution with marginal variances 0.4, 0.3 and correlation 0.65. Trait prevalence was 0.2 in both samples. π_0_, proportion of SNPs having no effect on traits. *P*, *P*-value for including SNP in the polygenic score. NCP, non-centrality parameter. Power computed at α = 0.05. AUC, area under receiver-operator characteristic curve. 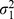, 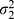, 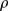, median estimates of model parameters, with coverage of 95%CI in brackets.(DOCX)

Table S3Simulations of binary traits in case/control samples compared to analytic results. Analytic values are in parentheses. Genotypes for 100,000 SNPs were simulated in 4000 subjects in each of two samples. Minor allele frequencies were drawn from Unif(0.01,0.5). Effect sizes on liability were drawn from a bivariate normal distribution with marginal variances 0.4, 0.3 and correlation 0.65. Trait prevalence was 0.001 in both samples, cases and controls sampled in equal proportion. π_0_, proportion of SNPs having no effect on traits. *P*, *P*-value for including SNP in the polygenic score. NCP, non-centrality parameter. Power computed at α = 0.05. AUC, area under receiver-operator characteristic curve. 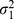, 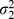, 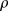, median estimates of model parameters, with coverage of 95%CI in brackets.(DOCX)

Table S4Simulations of quantitative traits compared to analytic results. Analytic values are in parentheses. Genotypes for 100,000 SNPs were simulated in 4000 subjects in each of two samples. Minor allele frequencies were drawn from Unif(0.01,0.5). Effect sizes in the two samples were drawn from Laplace distributions such that their marginal variances were 0.4, 0.3 and their correlation was 0.65. π_0_, proportion of SNPs having no effect on traits. *P*, *P*-value for including SNP in the polygenic score. NCP, non-centrality parameter. Power computed at α = 0.05. MSE, mean square error. 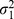, 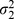, 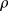, median estimates of model parameters, with coverage of 95%CI in brackets.(DOCX)

Table S5Simulations of binary traits in prospective samples compared to analytic results. Analytic values are in parentheses. Genotypes for 100,000 SNPs were simulated in 4000 subjects in each of two samples. Minor allele frequencies were drawn from Unif(0.01,0.5). Effect sizes on liability were drawn from Lapace distributions such that their marginal variances were 0.4, 0.3 and their correlation was 0.65. Trait prevalence was 0.2 in both samples. π_0_, proportion of SNPs having no effect on traits. *P*, *P*-value for including SNP in the polygenic score. NCP, non-centrality parameter. Power computed at α = 0.05. AUC, area under receiver-operator characteristic curve. 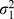, 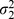, 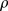, median estimates of model parameters, with coverage of 95%CI in brackets.(DOCX)

Table S6Simulations of binary traits in case/control samples compared to analytic results. Analytic values are in parentheses. Genotypes for 100,000 SNPs were simulated in 4000 subjects in each of two samples. Minor allele frequencies were drawn from Unif(0.01,0.5). Effect sizes on liability were drawn from Laplace distributions such that their marginal variances were 0.4, 0.3 and their correlation was 0.65. Trait prevalence was 0.001 in both samples, cases and controls sampled in equal proportion. π_0_, proportion of SNPs having no effect on traits. *P*, *P*-value for including SNP in the polygenic score. NCP, non-centrality parameter. Power computed at α = 0.05. AUC, area under receiver-operator characteristic curve. 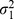, 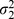, 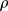, median estimates of model parameters, with coverage of 95%CI in brackets.(DOCX)

Text S1 Variance of genetic marker in a case/control sample.
(DOCX)

Text S2 Simulation of genotypes in a case/control study.
(DOCX)
