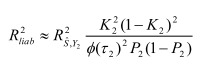# Correction: Power and Predictive Accuracy of Polygenic Risk Scores

**DOI:** 10.1371/annotation/b91ba224-10be-409d-93f4-7423d502cba0

**Published:** 2013-04-19

**Authors:** Frank Dudbridge

There are errors in equations and other expressions in the Methods section. In each instance in which a lowercase Greek script theta (ϑ) appears, it ought to be a lowercase Greek phi (ϕ). Please view the complete, correct equations here:

In the section titled "Binary traits":


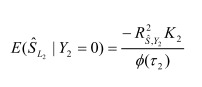



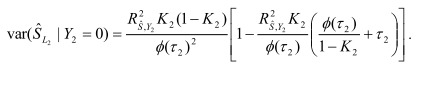


In the section titled “Case/Control Studies”:


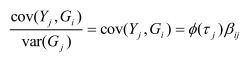



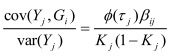



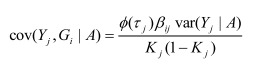



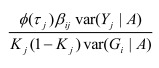



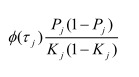



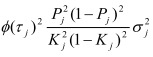



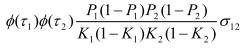



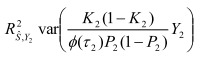



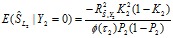


In the section titled “Liability R2”: