# Supplementary material for: Power and Predictive Accuracy of Polygenic Risk Scores
Source: PLoS Genet. 2013 Mar 21;9(3):e1003348. doi: 10.1371/journal.pgen.1003348 (PMC3605113; doi:10.1371/journal.pgen.1003348)
Supplement: Table S1 — Simulations of quantitative traits compared to analytic results. Analytic values are in parentheses. Genotypes for 100,000 SNPs were simulated in 4000 subjects in each of two samples. Minor allele frequencies were drawn from Unif(0.01,0.5). Effect sizes in the two samples were drawn from the bivariate normal distribution with marginal variances 0.4, 0.3 and correlation 0.65. π0, proportion of SNPs having no effect on traits. P, P-value for including SNP in the polygenic score. NCP, non-centrality parameter. Power computed at α = 0.05. MSE, mean square error. , , , median estimates of model parameters, with coverage of 95%CI in brackets. (DOCX) [file pgen.1003348.s001.docx]

Supplementary Table S1. Simulations of quantitative traits compared to analytic results. Analytic values are in parentheses. Genotypes for 100,000 SNPs were simulated in 4000 subjects in each of two samples. Minor allele frequencies were drawn from Unif(0.01,0.5). Effect sizes in the two samples were drawn from the bivariate normal distribution with marginal variances 0.4, 0.3 and correlation 0.65. π_0_, proportion of SNPs having no effect on traits. *P*, *P-*value for including SNP in the polygenic score. NCP, non-centrality parameter. Power computed at α=0.05. MSE, mean square error. $\sigma_{1}^{2}$, $\sigma_{2}^{2}$,$\rho$, median estimates of model parameters, with coverage of 95%CI in brackets.

|  |  | NCP | Power (%) | *R*^2^ | MSE | $\sigma_{1}^{2}$ (.4) | $\sigma_{2}^{2}$ (.3) | $\rho$ (.65) |
| --- | --- | --- | --- | --- | --- | --- | --- | --- |
| π_0_=0.95, *P*<0.1 | Linear regression | 5.618 (5.649) | 66.4 (66.16) | 0.00165 (0.00141) | 12.11 (12.11) | .4036 [94.9] | .3036 [94.9] | .6539 [94.9] |
|  | Shrinkage | 5.618 (5.649) | 66.4 (66.16) | 0.00165 (0.00141) | 1.606 (0.9988) | .4036 [94.9] | .3036 [94.9] | .6539 [94.9] |
|  | Allele count | 4.924 (4.916) | 60.70 (60.15) | 0.00148 (0.00123) | n/a | .4109 [95.4] | .3101 [95.4] | .6609 [95.4] |
| π_0_=0.99, *P*<10^-3^ | Linear regression | 24.66 (25.74) | 99.1 (99.91) | 0.00637 (0.00640) | 1.46 (1.363) | .3905 [89.6] | .2819 [89.6] | .6301 [89.6] |
|  | Shrinkage | 24.66 (25.74) | 99.1 (99.91) | 0.00637 (0.00640) | 1.167 (0.9984) | .3905 [89.6] | .2819 [89.6] | .6301 [89.6] |
|  | Allele count | 21.05 (22.12) | 98.8 (99.70) | 0.00548 (0.0055) | n/a | .3885 [90.4] | .2788 [90.4] | .6266 [90.4] |
