# Supplementary material for: Power and Predictive Accuracy of Polygenic Risk Scores
Source: PLoS Genet. 2013 Mar 21;9(3):e1003348. doi: 10.1371/journal.pgen.1003348 (PMC3605113; doi:10.1371/journal.pgen.1003348)
Supplement: Table S6 — Simulations of binary traits in case/control samples compared to analytic results. Analytic values are in parentheses. Genotypes for 100,000 SNPs were simulated in 4000 subjects in each of two samples. Minor allele frequencies were drawn from Unif(0.01,0.5). Effect sizes on liability were drawn from Laplace distributions such that their marginal variances were 0.4, 0.3 and their correlation was 0.65. Trait prevalence was 0.001 in both samples, cases and controls sampled in equal proportion. π0, proportion of SNPs having no effect on traits. P, P-value for including SNP in the polygenic score. NCP, non-centrality parameter. Power computed at α = 0.05. AUC, area under receiver-operator characteristic curve. , , , median estimates of model parameters, with coverage of 95%CI in brackets. (DOCX) [file pgen.1003348.s006.docx]

Supplementary Table S6. Simulations of binary traits in case/control samples compared to analytic results. Analytic values are in parentheses. Genotypes for 100,000 SNPs were simulated in 4000 subjects in each of two samples. Minor allele frequencies were drawn from Unif(0.01,0.5). Effect sizes on liability were drawn from Laplace distributions such that their marginal variances were 0.4, 0.3 and their correlation was 0.65. Prevalence was 0.001 for both traits, cases and controls sampled in equal proportion. π_0_, proportion of SNPs having no effect on traits. *P*, *P-*value for including SNP in the polygenic score. NCP, non-centrality parameter. Power computed at α=0.05. AUC, area under receiver-operator characteristic curve. $\sigma_{1}^{2}$, $\sigma_{2}^{2}$,$\rho$, median estimates of model parameters, with coverage of 95%CI in brackets.

|  |  | NCP | Power (%) | AUC (%) | $\sigma_{1}^{2}$ (.4) | $\sigma_{2}^{2}$ (.3) | $\rho$ (.65) |
| --- | --- | --- | --- | --- | --- | --- | --- |
| π_0_=0, *P*<1 | Linear regression | 25.78 (26.05) | 100.0 (99.91) | 54.51 (54.53) | .3959 [94.0] | .2970 [94.0] | .6467 [94.0] |
|  | Logistic regression | 25.14 (26.05) | 99.90 (99.91) | 54.50 (54.53) | .3851 [95.5] | .2891 [95.5] | .6381 [95.5] |
|  | Allele count | 16.17 (16.54) | 98.30 (98.25) | 53.58 (53.62) | .3892 [95.3] | .2921 [95.3] | .6414 [95.3] |
| π_0_=0.99, *P*<10^-3^ | Linear regression | 189.9 (173.6) | 100.0 (100.0) | 62.05 (61.41) | .4179 [75.9] | .3230 [75.9] | .6745 [75.9] |
|  | Logistic regression | 169.0 (173.6) | 100.0 (100.0) | 61.91 (61.41) | .3935 [83.7] | .2916 [83.7] | .6408 [83.7] |
|  | Allele count | 138.0 (144.0) | 100.0 (100.0) | 60.32 (60.44) | .3889 [82.3] | .2859 [82.3] | .6345 [82.3] |
