# Supplementary material for: Power and Predictive Accuracy of Polygenic Risk Scores
Source: PLoS Genet. 2013 Mar 21;9(3):e1003348. doi: 10.1371/journal.pgen.1003348 (PMC3605113; doi:10.1371/journal.pgen.1003348)
Supplement: Text S1 — Variance of genetic marker in a case/control sample. (DOCX) [file pgen.1003348.s007.docx]

## Text S1

## Variance of genetic marker in a case/control sample

Let $X\in\{0,1,2\}$ be the number of minor alleles at a biallelic marker with minor allele frequency $p$*,* and $G$ be its standardised value. Under Hardy-Weinberg Equilibrium (HWE) the population distribution of $G$ is

$$\left\{\begin{matrix} \frac{-2p}{\sqrt{2p(1-p)}}\text{ w.p. }(1-p)^{2} \\ \frac{1-2p}{\sqrt{2p(1-p)}}\text{ w.p. 2}p(1-p) \\ \frac{2-2p}{\sqrt{2p(1-p)}}\text{ w.p. }p^{2} \end{matrix}\right.$$

Let $\beta$ be the genetic effect on liability, $K$ be the trait prevalence, $\tau=\Phi^{-1}(1-K)=-\Phi^{-1}(K)$ and $Y=1$when liability exceeds $\tau$, 0 otherwise. The genotype frequencies in cases are

$$\Pr(X=0|Y=1)=\frac{\Pr(Y=1|X=0)\Pr(X=0)}{\Pr(Y=1)}=\frac{\Phi\left(\frac{-2p\beta}{\sqrt{2p(1-p)}}-\tau\right)(1-p)^{2}}{K}$$

$$\Pr(X=1|Y=1)=\frac{\Phi\left(\frac{\beta(1-2p)}{\sqrt{2p(1-p)}}-\tau\right)2p(1-p)}{K}$$

$$\Pr(X=2|Y=1)=\frac{\Phi\left(\frac{2\beta(1-p)}{\sqrt{2p(1-p)}}-\tau\right)p^{2}}{K}$$

Similarly the genotype frequencies in controls are

$$\Pr(X=0|Y=0)=\frac{\Phi\left(\tau+\frac{2p\beta}{\sqrt{2p(1-p)}}\right)(1-p)^{2}}{1-K}$$

$$\Pr(X=1|Y=0)=\frac{\Phi\left(\tau-\frac{\beta(1-2p)}{\sqrt{2p(1-p)}}\right)2p(1-p)}{1-K}$$

$$\Pr(X=2|Y=0)=\frac{\Phi\left(\tau-\frac{2\beta(1-p)}{\sqrt{2p(1-p)}}\right)p^{2}}{1-K}$$

The expectation of $G$ in the case/control sample is then

$$\begin{matrix} E(G|A)=\frac{-2p}{\sqrt{2p(1-p)}}\left(P\Pr(X=0|Y=1)+(1-P)\Pr(X=0|Y=0)\right)+ \\ \frac{1-2p}{\sqrt{2p(1-p)}}\left(P\Pr(X=1|Y=1)+(1-P)\Pr(X=1|Y=0)\right)+ \\ \frac{2-2p}{\sqrt{2p(1-p)}}\left(P\Pr(X=2|Y=1)+(1-P)\Pr(X=2|Y=0)\right)+ \end{matrix}$$

where $P$ is the sampling fraction of cases and $A$ denotes ascertainment, and the mean of $G^{2}$ is

$$\begin{matrix} E(G^{2}|A)=\frac{4p^{2}}{2p(1-p)}\left(P\Pr(X=0|Y=1)+(1-P)\Pr(X=0|Y=0)\right)+ \\ \frac{(1-2p)^{2}}{2p(1-p)}\left(P\Pr(X=1|Y=1)+(1-P)\Pr(X=1|Y=0)\right)+ \\ \frac{(2-2p)^{2}}{2p(1-p)}\left(P\Pr(X=2|Y=1)+(1-P)\Pr(X=2|Y=0)\right) \end{matrix}$$

The expected variance over a set of markers is obtained by integrating $\mathrm{var}(G|A)=E(G^{2}|A)-E(G|A)^{2}$ over the normal distribution of $\beta$. Values for a range of genetic variances and allele frequencies are given in tables A1 and A2. Except for scenarios in which rare markers explain a high proportion of liability, the variance of $G$ in the case/control sample is close to 1.

Table A1. Expected variance of genetic predictor in a case/control sample with equal number of cases and controls when the population prevalence is 0.1.

| $\mathrm{var}(G\vert A)$ | | $\mathrm{var}(\beta)$ | | | |
| --- | --- | --- | --- | --- | --- |
|  |  | 1/10 | 1/100 | 1/1000 | 1/10,000 |
| $p$ | 0.01 | 1.103 | 1.001 | 1.000 | 1.000 |
|  | 0.05 | 1.023 | 1.000 | 1.000 | 1.000 |
|  | 0.1 | 1.012 | 1.000 | 1.000 | 1.000 |
|  | 0.3 | 1.004 | 1.000 | 1.000 | 1.000 |
|  | 0.5 | 1.003 | 1.000 | 1.000 | 1.000 |

Table A2. Expected variance of genetic predictor in a case/control sample with equal number of cases and controls when the population prevalence is 0.01.

| $\mathrm{var}(G\vert A)$ | | $\mathrm{var}(\beta)$ | | | |
| --- | --- | --- | --- | --- | --- |
|  |  | 1/10 | 1/100 | 1/1000 | 1/10,000 |
| $p$ | 0.01 | 1.509 | 1.005 | 1.000 | 1.000 |
|  | 0.05 | 1.092 | 1.001 | 1.000 | 1.000 |
|  | 0.1 | 1.045 | 1.000 | 1.000 | 1.000 |
|  | 0.3 | 1.015 | 1.000 | 1.000 | 1.000 |
|  | 0.5 | 1.012 | 1.000 | 1.000 | 1.000 |
