# Supplementary material for: Power and Predictive Accuracy of Polygenic Risk Scores
Source: PLoS Genet. 2013 Mar 21;9(3):e1003348. doi: 10.1371/journal.pgen.1003348 (PMC3605113; doi:10.1371/journal.pgen.1003348)
Supplement: Text S2 — Simulation of genotypes in a case/control study. (DOCX) [file pgen.1003348.s008.docx]

## Text S2

## Simulation of genotypes in a case/control study

When disease liability is influenced by many thousands of markers, prospective simulation of case/control status is inefficient for rare disease. Retrospective simulation of genotypes under the liability threshold model is achieved as follows.

Let $X\in\{0,1,2\}$ be the number of minor alleles at a biallelic marker with minor allele frequency $p$under Hardy-Weinberg Equilibrium (HWE) and additive allelic effect $\beta$ on liability. Since liability is $\sim N(0,1)$, the mean liability for subjects with genotype $X$ is $\beta(X-2p)$, and assuming homoscedasticity its variance is $1-2p(1-p)\beta^{2}$. The probability of disease given genotype $X$ is therefore

$$\Pr(D=1|X)=1-\Phi(\tau;\beta(X-2p),1-2p(1-p)\beta^{2})$$

where $\tau=\Phi^{-1}(1-K)$ is the liability threshold for disease prevalence $K$. The frequency of genotype $X$ in cases is just

$$\Pr(X|D=1)=\frac{\Pr(D=1|X)p^{X}(1-p)^{2-X}2^{X\mathrm{mod}2}}{K}$$

Similarly

$$\Pr(D=0|X)=\Phi(\tau;\beta(X-2p),1-2p(1-p)\beta^{2})$$

and the frequency of genotype $X$ in controls is

$$\Pr(X|D=0)=\frac{\Pr(D=0|X)p^{X}(1-p)^{2-X}2^{X\mathrm{mod}2}}{1-K}$$

If all markers act additively upon liability, their genotypes remain independent in the ascertained sample and they may be simulated independently.
